# Expression profile of serum LncRNA THRIL and MiR-125b in inflammatory bowel disease

**DOI:** 10.1371/journal.pone.0275267

**Published:** 2022-10-07

**Authors:** Azza Elamir, Olfat Shaker, Marwa Kamal, Abeer Khalefa, Mostafa Abdelwahed, Fadwa Abd El Reheem, Tarek Ahmed, Essam Hassan, Shymaa Ayoub

**Affiliations:** 1 Medical Biochemistry and Molecular Biology Department, Faculty of Medicine, Fayoum University, Fayoum, Egypt; 2 Medical Biochemistry and Molecular Biology Department, Faculty of Medicine, Cairo University, Cairo, Egypt; 3 Department of Clinical Pharmacy, Faculty of Pharmacy, Fayoum University, Fayoum, Egypt; 4 Department of Physiology, Faculty of Medicine, Zagazig University, Zagazig, Egypt; 5 Department of Physiology, Faculty of Medicine, Fayoum University, Fayoum, Egypt; 6 Department of Clinical and Chemical Pathology, Faculty of Medicine, Fayoum University, Fayoum, Egypt; 7 Department of Internal Medicine, Faculty of Medicine, Fayoum University, Fayoum, Egypt; 8 Department of Tropical Medicine, Faculty of Medicine, Fayoum University, Fayoum, Egypt; Kunming University of Science and Technology, CHINA

## Abstract

**Background:**

Inflammatory bowel disease (IBD) is a chronic inflammatory disease of the gastrointestinal tract. We aimed to investigate, for the first time, the expression profile of serum level of LncRNA THRIL and MiR-125b in IBD patients and their relations with patient’s clinical and biochemical investigations.

**Methods:**

Our study included 210 subjects divided into 70 healthy subjects considered as control group (male and female), 70 patients with ulcerative colitis (UC), and 70 patients with Crohn’s disease (CD). Blood samples were obtained from all subjects. Expression of LncRNA THRIL and MiR-125b in serum was detected by Quantitative real time PCR (qRT-PCR).

**Results:**

Our results showed a significant increase in the fold change of LncRNA THRIL in UC patients (Median = 11.11, IQR; 10.21–12.45, P<0.001) and CD patients (Median = 5.87, IQR; 4.57–7.88, P<0.001) compared to controls. Meanwhile there was a significant decrease in the fold change of MiR-125b in UC patients (Median = 0.36, IQR; 0.19–0.61, P<0.001) and CD patients (Median = 0.69, IQR; 0.3–0.83, P<0.001) compared to controls. Furthermore, there was a negative significant correlation between LncRNA THRIL and MiR-125b in UC patients (r = -0.28, P = 0.016) and in CD patients (r = -0.772, P<0.001). ROC curve analysis was done showing the diagnostic value of these markers as predictors in differentiating between cases of UC, CD, and control.

**Conclusion:**

Serum LncRNA THRIL and MiR-125b could be used as potential biomarkers for diagnosis and prognosis of ulcerative colitis and Crohn’s disease.

## Introduction

Inflammatory bowel disease is a chronic inflammatory disease of the gastrointestinal tract that is classified into two types: ulcerative colitis (UC) and Crohn’s disease (CD), CD is characterized by patchy transmural inflammatory patterns of any portion along the intestinal wall affecting the whole thickness of the intestinal layers, While in case of UC the inflammatory process limited to large intestine affecting the innermost layers of the mucosa. The most frequent symptoms of these disorders are bloody diarrhea, abdominal pain, malabsorption, fatigue [1]. The condition could be complicated by intestinal fistula, intestinal obstruction, abdominal abscesses, and increased incidence of malignancy [2, 3]. Pathogenesis of IBD is still unclear but many factors interaction may play a role as genetic predisposition, microbial infection, and environmental factors [1].

Long non-coding RNAs (lncRNA) are transcripts) that contain more than 200 nucleotides and not translated into protein; they are processed by RNA polymerase II [4]. It was reported that lncRNA regulates cell function and biological processes in intestinal diseases such as irritable bowel syndrome [5], Hirschsprung’s disease [6], and IBD [7]. Many studies demonstrate the role of LncRNAs in IBD. For example, LncRNA CCAT1 promote IBD malignancy by downregulating miR-185-3p [8], LncRNA KIF9-AS1 promotes cell apoptosis by targeting the microRNA-148a-3p [9], LncRNA H19 act as a Competing Endogenous RNA to Regulate AQP Expression in the Intestinal Barrier of IBS-D Patients [10]. Haberman et al. reported 15 expressed LncRNAs differentially expressed in the tissue samples obtained from the patients with CD when compared to healthy control [11], another study by Wang et al. found that LINC01272 was highly expressed in peripheral blood and tissues of IBD [12]. The exact role of LncRNA is still unclear; it may act through chromatin remodeling, regulation of protein activity and its stability [13, 14].

LncRNA THRIL (TNFα and heterogeneous nuclear ribonucleoprotein L (hnRNPL) related immunoregulatory lincRNA) binds to the promoter region of the TNF-a forming RNA-protein complex inducing the expression of TNF-a, which contribute to the inflammatory process [15], and its dysregulation characterizes the autoimmune and inflammatory diseases. Chen et al also showed that knockdown of LncRNA THRIL decreases the levels of TNFa, IL1b, IL6, macrophages and neutrophils counts [16].

MicroRNA is a small single-stranded non-coding RNA molecule (containing about 22 nucleotides) that regulates gene expression through base pairing with complementary sequences within mRNA molecules [17]. Their abnormal activity has been demonstrated in many diseases including inflammatory and immunological disorders; Many studies reported that miRNA is associated with other pathophysiological factors of IBD, they may act by increasing or decreasing the intensity of the inflammatory process [18, 19], or by strengthening or weakening the intestinal barrier [20–23].

MiR-125b is transcribed from two loci located on chromosomes 11q23 (hsa-miR-125b-1) and 21q21 (hsa-miR-125b-2) [24]. It regulates the proliferation and differentiation of tumor cells and could be used as a diagnostic biomarker for early-stage cervical cancer and rheumatoid arthritis [25, 26].

The aim of this work was to evaluate the relative expression levels of serum LncRNA THRIL and MiR-125b in IBD patients and their relations with patients’ clinical and biochemical investigations.

## Materials and methods

### Subjects

Our study included 210 subjects divided into 70 healthy subjects considered as controls, 70 patients with ulcerative colitis, and 70 patients with Crohn’s disease. Patients were selected from outpatient clinics and inpatients of Tropical and Internal Medicine departments, Fayoum University Hospital, Fayoum University, Egypt. The study was revised and approved by the Ethical Committee of Faculty of Medicine, Fayoum University. Informed consent was obtained from all participants before sample collection.

The diagnosis of inflammatory bowel disease (IBD) with its 2 main subtypes, Crohn’s disease, and ulcerative colitis is based on patient history, clinical symptoms, radiological, and endoscopic criteria followed by histopathological examination of biopsies collected from each anatomic segment (rectum; sigmoid, left, transverse, and right colon; and ileum) according to European Crohn’s and Colitis Organization (ECCO) guidelines [27].

### The Crohn’s Disease Activity Index (CDAI)

CDAI was used for evaluating the disease severity of CD [28]. This index is scored on a scale from 0 to 1100 and includes abdominal pain, general wellbeing, complications, abdominal mass, anemia, and weight change. The patients with CD can be divided into

- Asymptomatic remission (CDAI < 150)
- Mild-to-moderate CD (150–220)
- Moderate-to-severe CD (220–450)
- Severe-fulminant disease (>450).

### The Mayo score

It was used for grading the severity of UC. The Score is based on four parameters: stool frequency, rectal bleeding, endoscopic findings, and Physician rating of disease activity [29]. Total criteria point count: Scores range from 0 to 12, with higher scores indicating more severe disease.

- Stool pattern: The patient reports a normal number of daily stools (0 points), 1 to 2 more stools than normal (1 point), 3 to 4 more stools (2 points), 5 or more stools (3 points).
- Rectal bleeding: None (0 points), Blood streaks seen in the stool less than half the time (1 point), all stools contain blood (2 points), Presence of pure blood (3 points).
- Endoscopic findings: Normal or inactive colitis seen (0 points), Mild colitis: mild erythema, decrease in vascularity (1 point), Moderate colitis: marked erythema, erosions seen (2 points), Severe colitis: spontaneous bleeding (3 points).
- Physician rating of disease activity: Normal (0 points), Mild colitis (1 point), Moderate colitis (2 points), severe colitis (3 points).

### Samples collection

Six-milliliter blood samples were drawn from all participants and collected in two tubes; one of them containing Ethylene Diamine TetraAcetic Acid (EDTA) for complete blood count (CBC) and Erythrocyte sedimentation rate (ESR) assay, the second tube left to clot for 15 min, centrifuged at 4000_g for 10 min, collect the serum, and stored at -80°C for all serological tests, including molecular biology techniques.

### RNA extraction

RNAs were extracted from serum using the miRNeasy Serum/Plasma Kit (Qiagen, Valenica, CA, USA) extraction kits following the manufacturer protocol, RNA concentration and purity were determined by Nano Drop2000 (Thermo Scientific, USA).

### Reverse transcription reactions

Reverse transcription was carried out on total RNA in a final volume of 20 μL(11 μl RNA + 2 μl genomic DNA elimination (GE)+ 7μl reverse-transcription mix). RNAs were reverse transcribed by RT-PCR kit into cDNAs using RvertAid First Strand cDNA Synthesis Kit (Thermo Fisher Scientific, USA) according to the manufacturer’s instructions.

### Quantitative real-time polymerase chain reaction

The cDNAs templates were amplified by Quantitative RT-PCR using the miScript SYBR Green PCR Kit (Qiagen, Germany). Regarding MiR-125b, the total volume was 25 μL per reaction using the specific MiR-125b primer (catalog numbers MS00006629 Lot. Number 20151214121). SNORD 68 was used to normalize the expression and for relative quantification of MiR-125b. While the total volume for LncRNA THRIL was 20 μL per reaction using the specific LncRNA THRIL primer (catalog numbers 330701LPH42418A) and GADPH was used to normalize the expression and for relative quantification of LncRNA THRIL. The qRT-PCR for both was programmed for the following cycling conditions: Initial activation step for 15 min at 95 °C then cycling denaturation for 15 sec at 94 °C, annealing for 30 sec at 55 °C, and extension for 30 sec at 70 °C, and these steps were repeated for 40 cycles in Rotor-gene qRT-PCR system thermocycler (Qiagen, USA). The relative expression of RNAs was calculated by the 2^-ΔΔCt^ method [30].

### Statistical analysis

The collected data were arranged and statistically analyzed using SPSS software with statistical computer package version 25 (SPSS). For quantitative data, the mean, median, SD, and interquartile range were calculated. If the variable was not normally distributed, the Mann–Whitney U test or the Kruskal–Wallis test was used for comparison between any two groups or three groups, respectively. Otherwise, one-way ANOVA was used. Qualitative data were presented as number and percentages. Chi-square (χ2) was used as a test of significance. Spearman’s correlation was run to identify the relation of LNC THRIL and MiR-125b with study parameters. (ROC) curves were used to determine the cutoff point, which shows the highest sensitivity and specificity of LNC THRIL and MiR-125b in differentiating between different study groups. P values <0.05 were considered as statistically significant.

## Results

### Demographic, clinical and laboratory characteristics of the study groups

There was no significant difference between patients and controls as regards age (p = 0.106) and sex (P = 0.868). Results showed a highly statistically significant difference between UC, CD, and control groups as regards Hb (P<0.001), Total leucocytic count (P<0.001), Platelets number (P<0.001) with high level in CD and Albumin level (P<0.001) with a low level in CD. There was also a highly statistically significant difference between UC, CD groups as regards incidence of diabetes (P = 0.004), musculoskeletal complications (P = 0.006), Hematocrit (P = 0.041), CRP (P <0.001) and ESR (P = 0.023) with high level in CD group and as regards patients on salicalyates (P = 0.002) with a high level in UC (Table 1).

**Table 1:** Demographic, biochemical and clinical characteristics of the study groups.

		Ulcerative colitis		Crohn’s Disease		Control		P-value
		Mean	SD	Mean	SD	Mean	SD	
Age		32.1	3.7	33.2	1.7	30	2.3	0.106
Duration of illness		3.8	0.3	4.6	0.1	.	.	0.436
		No	%	No	%	No	%	
sex	Female	28	40.0%	26	37.1%	25	35.7%	0.868
	male	42	60.0%	44	62.9%	45	64.3%	
smoker	no	54	77.1%	48	68.6%	59	84.3%	0.089
	yes	16	22.9%	22	31.4%	11	15.7%	
coffee consumption	no	58	82.9%	48	68.6%	54	77.1%	0.136
	yes	12	17.1%	22	31.4%	16	22.9%	
diabetes	no	62	88.6%	68	97.1%			0.004*
	yes	8	11.4%	2	2.9%			
hypertension	no	68	97.1%	68	97.1%			0.361
	yes	2	2.9%	2	2.9%			
Other comorbidities	congenital heart disease (TS)	0	0.0%	2	2.9%			
	Ischemic heart disease	0	0.0%	2	2.9%			
	stroke	0	0.0%	2	2.9%			
	thyrotoxic	2	2.9%	0	0.0%			
	no	68	97.1%	64	91.4%			
**Extraintestinal**								
Hepatobiliary		6	8.6%					
Endocrine		2	2.9%					
Arthralgia				4	5.7%			
Thromboembolic				10	14.3%			
MSK		22	31.4%	38	54.3%			0.006*
Eye		10	14.3%	8	11.4%			0.614
**History (Crohn’s)**								
ileal strictures				10	4.8%			
rectal fistula				20	9.5%			
Perforation				8	3.8%			
Intestinal obstruction				12	5.7%			
surgical resection				8	3.8%			
colocutaneous fistula				8	3.8%			
perianal abcess or fistula				4	1.9%			
colonic stricture				4	1.9%			
psoas abcess				6	2.9%			
**Treatment**								
Salicalyates		42	60.0%	24	34.3%			0.002*
Steroids		38	54.3%	30	42.9%			0.176
Azathioprine		18	25.7%	20	28.6%			0.704
Infliximab		10	14.3%	16	22.9%			0.192
Mayo score		7.6	3.6	.	.	.	.	
CDAI		.	.	267.3	130.9	.	.	
Hb gl/dl		11.2	2	12.9	2	12	1.2	<0.001*
HCT		34.4	6.1	36.5	5.9	.	.	0.041*
TLC		8.3	4.8	8.5	3.9	5.5	1.9	<0.001*
Neutrophil count		60.7	16.6	60.5	12.1	.	.	0.936
Platelets		317.3	107.4	348.2	126	210.1	34.8	<0.001*
CRP		14.2	12.2	26.8	26.1	.	.	<0.001*
ESR		26.5	18.5	35.5	27.3	.	.	0.023*
Albumin		3.7	0.8	3.6	0.7	4.6	0.3	<0.001*

### Description of fold change of MiR-125b and Lnc THRIL among study groups

Results showed significant differences between the patients’ groups and control group regarding Lnc THRIL and MiR-125b with the fold change of Lnc THRIL was significantly up-regulated in UC patients (Median = 11.11, IQR; 10.21–12.45, P<0.001) and CD patients (Median = 5.87, IQR; 4.57–7.88, P<0.001) compared to controls. Meanwhile, the fold change of MiR-125b was significantly down-regulated in UC patients (Median = 0.36, IQR; 0.19–0.61, P<0.001) and CD patients (Median = 0.69, IQR; 0.3–0.83, P<0.001) compared to controls (Table 2).

**Table 2:** Description of fold change of MiR-125b and Lnc THRIL among study groups.

	Ulcerative colitis			Crohn’s			Control		
	Median	IQR		Median	IQR		Median	IQR	
**MiR-125b**	0.36	0.19	0.61	0.69	0.31	0.83	1	1	1
**P-values**	0.004*								
				<0.001*					
	<0.001*								
**Lnc THRIL**	11.11	10.21	12.45	5.87	4.57	7.88	1	1	1
**P-values**	<0.001*								
				<0.001*					
	<0.001*								

### Relations between Lnc THRIL and MiR-125b and clinical data in UC and CD patients

We found that the level of LncRNA THRIL was significantly higher in nonsmoker UC patients than in smokers (P = 0.012) and the level of MiR-125b was significantly higher in patients with endocrinal complication (P = 0.041) (Table 3). Meanwhile in CD patients, results showed that the level of LncRNA THRIL was significantly higher in females (P <0.001), nondiabetic patients (P <0.001), patients with intestinal perforation (P = 0.002), and patients with colonic stricture (P = 0.026). As regards MiR-125b, it was significantly low in females (P = 0.002), patients on salicylates (P = 0.040), patients with colonic stricture (P = 0.002) and significantly high in patients with thromboembolic complications (P = 0.007), musculoskeletal complications (P = 0.021), rectal fistula (P = 0.002) and perforation (P = 0.001) (Table 4).

**Table 3:** Relations between Lnc THRIL and MiR-125b and clinical data in UC patients.

		miR-125b				Lnc THRIL			
		Median	IQR		P-value	Median	IQR		P-value
Sex	Female	0.27	0.18	0.6	0.208	10.89	10.35	11.4	0.414
	male	0.49	0.23	0.63		11.12	10.21	12.45	
Smoker	yes	0.33	0.22	0.57	0.911	10.21	10.18	10.88	0.012*
	no	0.41	0.19	0.61		11.19	10.76	12.45	
Coffee consumption	yes	0.34	0.12	0.51	0.417	11.78	11.05	12.53	0.109
	no	0.41	0.21	0.63		10.94	10.18	12.29	
Diabetes	yes	0.57	0.31	0.67	0.284	10.18	9.46	11.75	0.159
	no	0.34	0.19	0.6		11.12	10.21	12.45	
Hypertension	yes	0.3	0.28	0.31	0.720	11.05	11	11.11	0.902
	no	0.39	0.19	0.62		11.12	10.19	12.45	
Hepatobiliary	yes	0.29	0.11	0.36	0.101	12.29	8.25	12.45	0.976
	no	0.45	0.2	0.63		11.06	10.21	12.37	
Endocrine	yes	0.73	0.7	0.76	0.041*	10.38	10	10.76	0.239
	no	0.35	0.19	0.61		11.12	10.21	12.45	
MC	yes	0.62	0.59	0.64	0.225	11.02	10.7	11.35	0.901
	no	0.35	0.19	0.61		11.11	10.19	12.45	
MSK	yes	0.34	0.23	0.55	0.586	10.79	10.18	11.4	0.054
	no	0.43	0.18	0.64		11.12	10.76	12.49	
Eye	yes	0.23	0.23	0.41	0.202	11.12	10.89	11.4	0.788
	no	0.43	0.19	0.64		11.06	10.19	12.45	
Salicalyates	yes	0.49	0.23	0.64	0.179	10.94	10.18	11.4	0.358
	no	0.32	0.18	0.57		11.19	10.21	12.45	
Steroids	yes	0.3	0.18	0.63	0.662	11.11	10.18	12.45	0.482
	no	0.45	0.24	0.59		11.07	10.76	12.37	
Azathioprine	yes	0.5	0.23	0.63	0.404	11.01	10.18	11.12	0.194
	no	0.33	0.18	0.61		11.12	10.21	12.45	
Infliximab	yes	0.31	0.19	0.64	0.626	11.12	10.18	11.35	0.699
	no	0.39	0.21	0.61		11.06	10.21	12.45	

**Table 4:** Relations between Lnc THRIL and MiR-125b and clinical data in CD patients.

		miR-125b				Lnc THRIL			
		Median	IQR		P-value	Median	IQR		P-value
Sex	Female	0.31	0.27	0.78	0.002*	7.88	5.77	8.23	<0.001*
	male	0.78	0.4	0.92		5.53	4.43	6.47	
Smoker	yes	0.69	0.22	0.96	0.693	5.87	4.43	7.21	0.510
	no	0.67	0.31	0.78		5.9	4.67	7.97	
Coffee consumption	no	0.62	0.25	0.8	0.394	5.55	4.43	6.89	0.100
	yes	0.74	0.4	0.83		6.02	4.81	7.97	
Diabetes	yes	0.74	0.7	0.8	0.720	2.07	2	2.13	<0.001*
	no	0.67	0.3	0.83		5.95	4.76	7.88	
Hypertension	yes	0.37	0.35	0.4	0.518	5.21	5	5.42	0.438
	no	0.7	0.3	0.83		5.95	4.57	7.88	
Arthralgia	yes	0.59	0.4	0.78	1.000	5.76	5.5	6.02	0.817
	no	0.69	0.3	0.83		5.87	4.57	7.88	
MSK	yes	0.69	0.3	0.83	0.021*	8.12	5.42	9.01	0.066
	no	0.30	0.19	0.35		5.82	4.56	7.54	
Thromboembolic	yes	0.78	0.31	0.83	0.007*	7.21	5.66	8.23	0.070
	no	0.31	0.23	0.4		5.82	4.54	7.65	
MC	yes	0.78	0.31	0.83	0.585	5.55	4.54	7.65	0.383
	no	0.78	0.4	0.92		5.95	5.46	7.9	
Eye	yes	0.4	0.25	0.78	0.363	5.82	4.65	7.5	0.912
	no	0.71	0.47	1.26		5.87	4.57	7.88	
Salicalyates	yes	0.69	0.27	0.83	0.040*	5.23	4.5	7.06	0.166
	no	0.81	0.4	0.94		6.02	4.76	8	
Steroids	yes	0.64	0.23	0.78	0.311	5.5	4.57	7.88	0.962
	no	0.54	0.3	0.96		5.95	4.55	7.68	
Azathioprine	yes	0.7	0.27	0.78	0.214	5.97	4.57	7.65	0.959
	no	0.66	0.4	0.96		5.87	4.76	7.88	
Infliximab	yes	0.69	0.3	0.78	0.456	5.79	4.67	7.39	0.978
	no	0.78	0.43	0.85		5.87	4.43	7.93	
Ileal strictures	yes	0.78	0.78	0.96	0.098	5.87	4.76	6.47	0.946
	no	0.59	0.3	0.78		5.9	4.57	7.88	
Rectal fistula	yes	0.81	0.64	0.96	0.002*	5.39	4.43	6.47	0.145
	no	0.40	0.23	0.78		6.02	4.76	7.93	
Perforation	yes	1.08	0.76	1.53	0.001*	6.02	5.05	7.93	0.002*
	no	0.54	0.27	0.78		4.27	3.28	5.1	
Intestinal obstruction	yes	0.78	0.31	0.83	0.562	4.55	4.11	7.93	0.152
	no	0.64	0.3	0.78		6.02	5.05	7.65	
Surgical resection	yes	0.8	0.5	0.83	0.383	4.44	4.05	6.6	0.121
	no	0.64	0.3	0.78		6.02	5.05	7.88	
Colocutaneous fistula	yes	0.57	0.33	0.78	0.578	5.9	3.95	7.23	0.853
	no	0.69	0.3	0.83		5.87	4.57	7.88	
Perianal abcess or fistula	yes	0.45	0.12	0.78	0.229	6.11	4.33	7.88	0.779
	no	0.69	0.31	0.83		5.87	4.76	7.65	
Colonic stricture	yes	0.17	0.12	0.23	0.002*	8.06	7.88	8.23	0.026*
	no	0.74	0.31	0.83		5.77	4.57	7.43	
Psoas abcess	yes	0.74	0.22	0.83	0.959	4.11	2.13	8.44	0.188
	no	0.67	0.31	0.8		5.95	4.88	7.77	

### Correlations of Lnc THRIL and MiR-125b with study parameters among the patients

In UC patients, our results showed that there were negative significant correlations between LncRNA THRIL and each of MiR-125b (r = -0.28, P = 0.016), duration of illness (r = -0.35, P = 0.020), and ESR (r = -0.24, P = 0.042) and between MiR-125b and both ESR(r = -0.38, P = 0.001) and neutrophil count (r = -0.24, P = 0.039) (Table 5). In CD patients, our results showed that there were negative significant correlations between LncRNA THRIL and each of MiR-125b (r = -0.77, P<0.001), age (r = -0.23, P = 0.048), TLC (r = -0.41, P<0.001), neutrophil count (r = -0.42, P<0.001), platelets (r = -0.29, P = 0.014), and CRP (r = -0.35, P = 0.002), and positive significant correlations between MiR-125b and both TLC (r = 0.37, P = 0.001) and platelets (r = -0.29, P = 0.012) (Table 6).

**Table 5:** Correlations of Lnc THRIL and MiR 125b with study parameters among UC patients.

		MiR-125b	Lnc THRIL
Lnc THRIL	r	**- 0.288**	
	P-value	0.016*	
Age	r	0.013	-0.054
	P-value	0.913	0.659
Duration of illness	r	0.148	**-0.357**
	P-value	0.351	0.020*
Hb gl/dl	r	-0.033	0.023
	P-value	0.788	0.847
HCT	r	-0.020	0.055
	P-value	0.869	0.651
TLC	r	-0.183	-0.225
	P-value	0.129	0.061
Neutrophil count	r	**-0.248**	0.076
	P-value	0.039*	0.531
Platelets	r	0.020	-0.185
	P-value	0.869	0.125
CRP	r	-0.201	-0.221
	P-value	0.095	0.066
ESR	r	**-0.380**	**-0.243**
	P-value	0.001*	0.042*
Albumin(mg/dl)	r	0.076	0.049
	P-value	0.533	0.688
Mayo score	r	-0.170	-0.210
	P-value	0.159	0.081

**Table 6:** Correlations of Lnc THRIL and MiR 125b with study parameters among CD patients.

		MiR-125b	Lnc THRIL
Lnc THRIL	r	**-0.772**	
	P-value	<0.001*	
Age	r	0.016	**-0.237**
	P-value	0.893	0.048*
Duration of illness	r	-0.134	0.024
	P-value	0.387	0.876
Hb gl/dl	r	0.139	-0.048
	P-value	0.252	0.695
HCT	r	0.019	0.052
	P-value	0.874	0.667
TLC	r	**0.378**	**-0.416**
	P-value	0.001*	<0.001*
Neutrophil count	r	0.165	**-0.428**
	P-value	0.171	<0.001*
Platelets	r	**0.299**	**-0.293**
	P-value	0.012*	0.014*
CRP	r	0.109	**-0.359**
	P-value	0.371	0.002*
ESR	r	-0.024	-0.138
	P-value	0.844	0.256
Albumin	r	-0.129	0.189
	P-value	0.289	0.118
CDAI	r	0.195	-0.130
	P-value	0.105	0.285

### Receiver operating characteristic of sensitivity and specificity of Lnc THRIL and MiR-125b in the study groups

**Fig 1:**
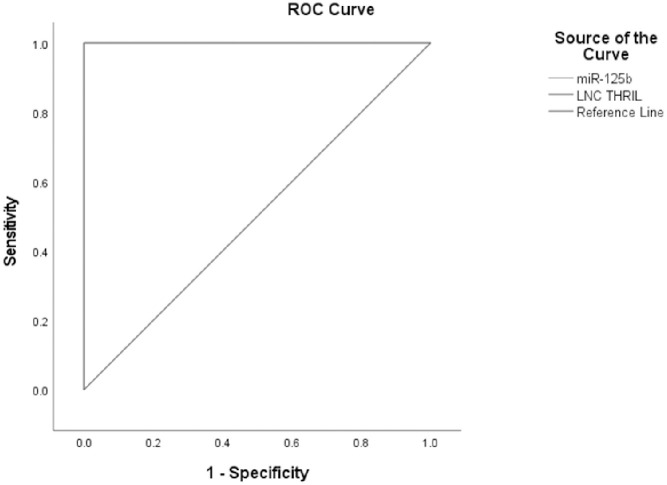
Receiver operating characteristic of sensitivity and specificity of Lnc THRIL and MiR-125b in UC patients vs. controls.

Fig 1 illustrates the ROC curve of Lnc THRIL and MiR-125b in the UC group, showing the diagnostic value of these markers as predictors in differentiating between cases of UC and control. Lnc THRIL; AUC = 1.000, P<0.0001, cut off point 4.62, sensitivity 100%, specificity 100.0%. MiR-125b; AUC = 1.000, P<0.0001, cut off point 0.94, sensitivity 100%, specificity 100.0%.

**Fig 2:**
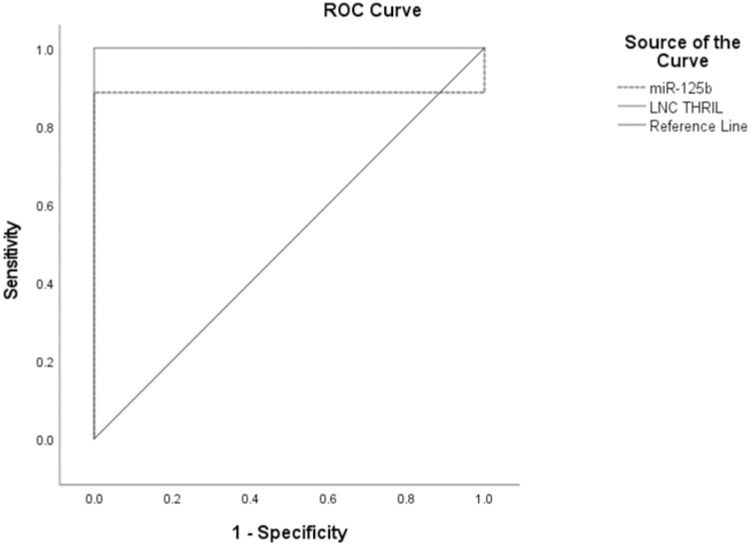
Receiver operating characteristic of sensitivity and specificity of Lnc THRIL and MiR-125b in CD patients vs. controls.

Fig 2 illustrates the ROC curve of Lnc THRIL and MiR-125b in the CD group, showing the diagnostic value of these markers as predictors in differentiating between cases of CD and control. Lnc THRIL; AUC = 1.000, P<0.0001, cut off point 1.57, sensitivity 100%, specificity 100.0%. MiR-125b; AUC = 0.886, P<0.0001, cut off point 0.98, sensitivity 88.6%, specificity 100.0%.

**Fig 3:**
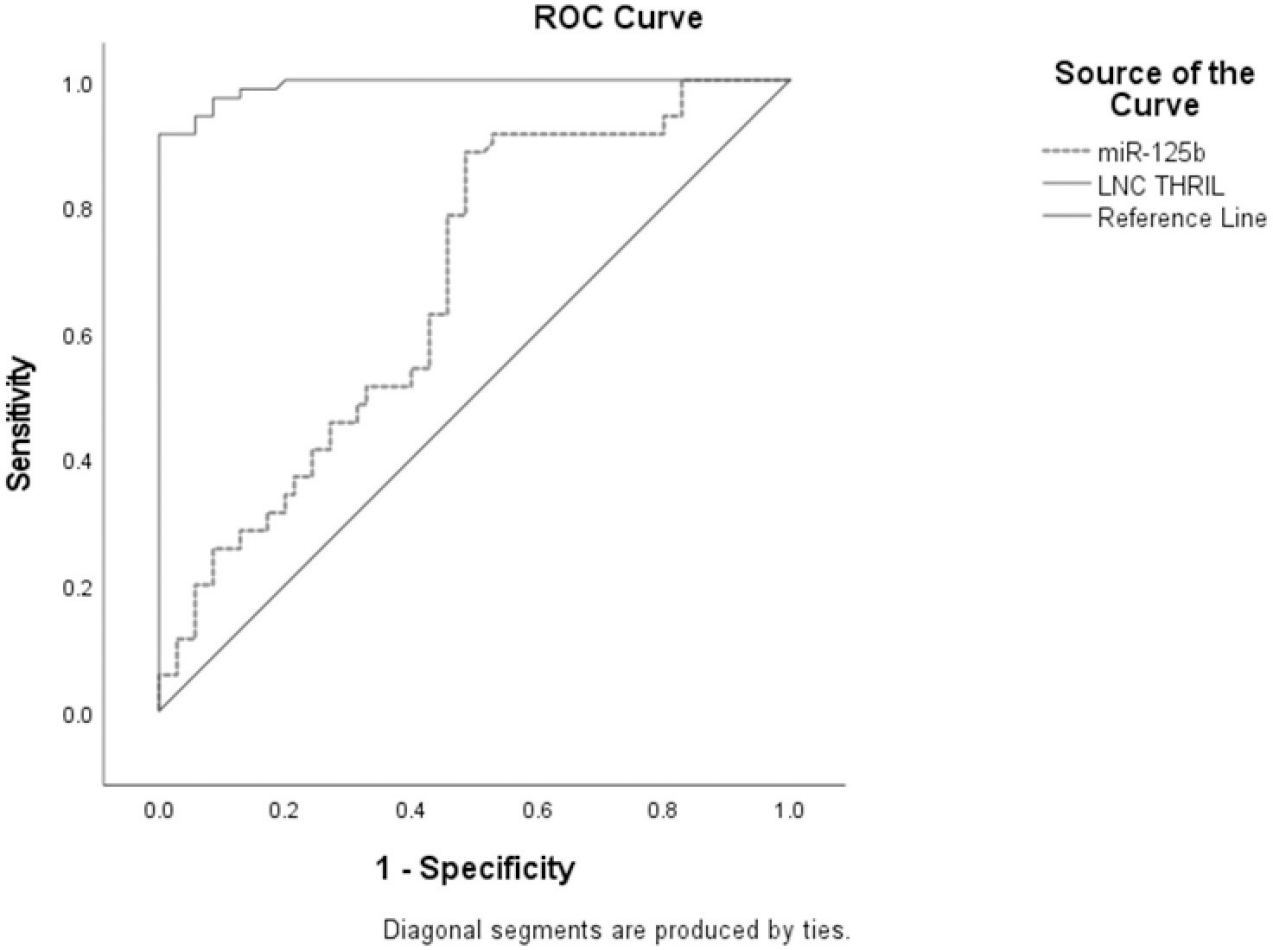
Receiver operating characteristic of sensitivity and specificity of Lnc THRIL and MiR-125b in UC vs. CD patients.

Fig 3 illustrates the ROC curve of Lnc THRIL and MiR-125b showing the diagnostic value of these markers as predictors in differentiating between CD and UC cases. Lnc THRIL; AUC = 0.992, P<0.0001, cut off point 9.49, sensitivity 91.4%, specificity 100.0%. MiR-125b; AUC = 0.677, P<0.0001, cut off point 0.67, sensitivity 88.6%, specificity 51.4%.

## Discussion

Inflammatory bowel disease (IBD) is characterized by repetitive episodes of inflammation of the gastrointestinal tract, its exact cause remains unclear, knowing the pathogenesis of IBD will help to develop new strategies for therapies and reduce the incidence of complications. Interaction between the gastrointestinal microbiome and host immune system has been evidenced as a cause [31].

The role of non-coding RNAs (ncRNAs) has been reported in IBD. NcRNAs include microRNAs (miRNAs), long noncoding RNAs (lncRNAs), and circular RNAs (circRNAs) [32, 33]. Our study focused on the expression profile of LncRNA THRIL and MiR-125b in IBD and their relation with patient’s clinical and biochemical investigations.

THRIL (TNF- α and hnRNPL immunoregulatory lncRNA) is involved in innate immunity through the regulation of TNF -α expression level by forming a complex that bind TNF- α gene promotor region resulting in its induction, TNF- α is one of the cytokines that collaborate in the inflammatory process and its dysregulation characterizes autoimmune diseases [34]. It is implicated in a wide range of cellular processes including cell proliferation, survival, and death. In addition, TNF-α signaling is associated with the regulation of several inflammatory pathways including the cyclooxygenase-2 (COX-2) and induce nitric oxide synthase (iNOS) pathways [35]. Several studies investigating the role of anti-TNF-α therapy in modulating the consequences of IBD by different mechanisms as inhibiting activation of immune cells [36], downregulates the expression of cell adhesion molecules and proinflammatory cytokines [37] and has a favorable effect on the gut microbiome [38].

Our study showed a significant difference between the patients’ groups and control group regarding Lnc THRIL with the fold change of Lnc THRIL was significantly up-regulated in UC patients (Median = 11.11, IQR; 10.21–12.45, P<0.001) and CD patients (Median = 5.87, IQR; 4.57–7.88, P<0.001) compared to controls. As regards MiR-125b. Our study showed that the fold change of MiR-125b was significantly down-regulated in UC patients (Median = 0.36, IQR; 0.19–0.61, P<0.001) and CD patients (Median = 0.69, IQR; 0.3–0.83, P<0.001) compared to controls, This dysregulation could be explained by that TRAF6(TNF receptor associated factor 6) and TNFAIP3 (TNF alpha induced protein 3) also referred to as A20 are the two key signaling molecules involved in the NFκB pathway, and these genes carry complementary binding sites for miR-125b in their 3′UTRs and the role of NFκB pathway activation in several autoimmune diseases including IBD and various cancers was previously confirmed [39].

Increased NF-κB expression in mucosal macrophages increases the levels of pro-inflammatory cytokines such as TNF-α, IL-1 and IL-6 resulting in the mucosal cells damage, and it increases expression of intercellular adhesion molecule-1 in colonic epithelial cells that contributes to the recruitment of neutrophil granulocytes to the site of inflammation [40]. This imbalance between excessive secretion of pro-inflammatory cytokines and relative insufficient secretion of anti-inflammatory cytokines is linked to the development of non-specific inflammatory responses in the intestine [41]. Our data disagreed with a report done by Valmiki S et al who showed that MiR125b was significantly up-regulated in UC patients as compared to controls [42].

Moreover, The study showed that there was a negative significant correlation between LncRNA THRIL and MiR-125b in both UC(r = -0.28, P = 0.016) and CD patients(r = -0.77, P<0.001), This finding was previously confirmed by Liu et al who showed that Long non-coding RNA THRIL promotes lipopolysaccharide (LPS) induced inflammatory injury by down-regulating microRNA-125b in ATDC5 cells which act as cell line model of cartilage extracellular matrix neosynthesis and maturation [43]. Song et al., also showed that LncRNA THRIL expression was negatively correlated with miR-125b expression in allergic rhinitis patients [44]. Several studies demonstrated that lncRNAs can exert many cellular functions by interacting with miRNAs as inhibitors or RNA decoys to reduce miRNA production and availability. These interactions play an important role in intestinal epithelial homeostasis [45]. We detected negative significant correlations between MiR-125b and both ESR and neutrophil count in UC patients which agreed with Hruskova et al., who observed negative correlation between expression of miR-125b and the parameters of disease activity and detected inverse correlation between miR-125b and ESR (r = -0.268, P = 0.042) [46]. This finding supports miR-125b’s inhibitory effect on the expression of pro-inflammatory cytokines, cell proliferation, and apoptosis [47].

We also detected positive significant correlations between MiR-125b and both TLC and platelets in CD patients, which agreed with Marina et al., who showed that Hematopoietic cells benefit from miR-125b overexpression in terms of proliferation and CBC results obtained 16 weeks posttransplant revealed an increase in WBC in mice expressing miR-125b compared to control mice. There were statistically significant increases in neutrophils in particular [48].

## References

[1] Guan Q. (2019). A Comprehensive Review and Update on the Pathogenesis of Inflammatory Bowel Disease. J Immunol Res. doi: 10.1155/2019/7247238 . 31886308 PMC6914932

[2] Stidham RW, Higgins PDR. (2018) Colorectal Cancer in Inflammatory Bowel Disease. Clin Colon Rectal Surg. 31: 168–783. doi: 10.1055/s-0037-1602237 29720903 PMC5929884

[3] Habibi F, Habibi ME, Gharavinia A, et al. (2017) Quality of life in inflammatory bowel disease patients: a cross-sectional study. J Res Med Sci. 22:104. doi: 10.4103/jrms.JRMS_975_16 29026420 PMC5629832

[4] Chen L. L. (2016). Linking long noncoding RNA localization and function. Trends Biochem. Sci. 41, 761–772. doi: 10.1016/j.tibs.2016.07.003 27499234

[5] Zhang Y, Zhang H, Zhang W, Zhang Y, Wang W, Nie L. (2020). LncRNA XIST modulates 5-hydroxytrytophan-induced visceral hypersensitivity by epigenetic silencing of the SERT gene in mice with diarrhea-predominant IBS. Cell Signal. 73:109674. doi: 10.1016/j.cellsig.2020.109674 32446903

[6] Cai P, Li H, Huo W, Zhu H, Xu C, Zang R, et al. (2018). Aberrant expression of LncRNA-MIR31HG regulates cell migration and proliferation by affecting miR-31 and miR-31\ in Hirschsprung’s disease. J Cell Biochem; 119:8195–8203. doi: 10.1002/jcb.26830 29626357

[7] Lucafò M, Di Silvestre A, Romano M, Avian A, Antonelli R, Martelossi S, et al. (2018). Role of the long non-coding RNA growth arrest-specific 5 in glucocorticoid response in children with inflammatory bowel disease. Basic Clin Pharmacol Toxicol. 122:87–93. doi: 10.1111/bcpt.12851 28722800

[8] Ma D, Cao Y, Wang Z, He J, Chen H, Xiong H et al.(2019).CCAT1 lncRNA Promotes Inflammatory Bowel Disease Malignancy by Destroying Intestinal Barrier via Downregulating miR-185-3p. Inflamm Bowel Dis. Apr 11; 25(5):862–874. doi: 10.1093/ibd/izy381 30615124

[9] Yao J, Gao R, Luo M, Li D, Guo L, Yu Z et al.(2021). Long noncoding RNA KIF9-AS1 promotes cell apoptosis by targeting the microRNA-148a-3p/suppressor of cytokine signaling axis in inflammatory bowel disease. Eur J Gastroenterol Hepatol. Epub ahead of print. doi: 10.1097/MEG.0000000000002309 . 34750325 PMC8734634

[10] Chao G, Wang Z, Yang Y and Zhang S (2021) LncRNA H19 as a Competing Endogenous RNA to Regulate AQP Expression in the Intestinal Barrier of IBS-D Patients. Front. Physiol. 11:602076. doi: 10.3389/fphys.2020.602076 33584332 PMC7874183

[11] Haberman Y., BenShoshan M., di Segni A. et al., (2018) Long ncRNA landscape in the ileum of treatment-naive early-onset Crohn disease. Inflammatory Bowel Diseases, vol. 24, no. 2, pp. 346–360,. doi: 10.1093/ibd/izx013 29361088 PMC6231367

[12] Wang S., Hou Y., Chen W. et al. (2018) “KIF9-AS1, LINC01272 and DIO3OS lncRNAs as novel biomarkers for inflammatory bowel disease,” Molecular Medicine Reports, vol. 17(2): 2195–2202,. doi: 10.3892/mmr.2017.8118 29207070 PMC5783463

[13] Wu F., Huang Y., Dong F. & Kwon J. H. (2016). Ulcerative colitis-associated long noncoding RNA, BC012900, regulates intestinal epithelial cell apoptosis. Inflamm. Bowel Dis. 22, 782–795. 10.1097/MIB.0000000000000691 26937624

[14] Quinn J. J. & Chang H. Y. (2016). Unique features of long non-coding RNA biogenesis and function. Nat. Rev. Genet. 17, 47–62. doi: 10.1038/nrg.2015.10 26666209

[15] Li Z, Chao TC, Chang KY, et al. (2014). The long noncoding RNA THRIL regulates TNFa expression through its interaction with hnRNPL. Proc Natl Acad Sci U S A. 111 (3):1002–1007. 24371310 10.1073/pnas.1313768111 PMC3903238

[16] Chen H, Hu X, Li R, et al. (2020).LncRNA THRIL aggravates sepsis induced acute lung injury by regulating miR-424/ROCK2 axis. Mol Immunol.;126:111–119. doi: 10.1016/j.molimm.2020.07.021 32818819

[17] Chen PY, Meister G. (2005): MicroRNA-guided posttranscriptional gene regulation. Biol Chem. 386:1205–1218. doi: 10.1515/BC.2005.139 16336116

[18] Zhao Y, Ma T, Chen W, Chen Y, Li M, Ren L, et al. (2016). MicroRNA-124 Promotes Intestinal Inflammation by Targeting Aryl Hydrocarbon Receptor in Crohn’s Disease. J Crohns Colitis. 10: 703–12. doi: 10.1093/ecco-jcc/jjw010 26802080

[19] Pierdomenico M, Cesi V, Cucchiara S, Vitali R, Prete E, Costanzo M, et al. (2016).NOD2 Is Regulated By Mir-320 in Physiological Conditions but this Control Is Altered in Inflamed Tissues of Patients with Inflammatory Bowel Disease. Inflamm Bowel Dis. 22: 315–26. doi: 10.1097/MIB.0000000000000659 26752466

[20] Shen Y, Zhou M, Yan J, Gong Z, Xiao Y, Zhang C, et al. (2017). miR-200b inhibits TNF-alpha-induced IL-8 secretion and tight junction disruption of intestinal epithelial cells in vitro. Am J Physiol Gastrointest Liver Physiol. 312:G123–G32. 27979826 10.1152/ajpgi.00316.2016

[21] Haines RJ, Beard RS Jr., Eitner RA, Chen L, Wu MH. (2016). TNFalpha/IFNgamma Mediated Intestinal Epithelial Barrier Dysfunction Is Attenuated by MicroRNA-93 Downregulation of PTK6 in Mouse Colonic Epithelial Cells. PLoS One.; 11: e0154351. 27119373 10.1371/journal.pone.0154351 PMC4847919

[22] Zou T, Jaladanki SK, Liu L, Xiao L, Chung HK, Wang JY, et al. (2016). H19 Long Noncoding RNA Regulates Intestinal Epithelial Barrier Function via MicroRNA 675 by Interacting with RNA-Binding Protein HuR. Mol Cell Biol. 36: 1332–1341. doi: 10.1128/MCB.01030-15 26884465 PMC4836219

[23] Zhang B, Tian Y, Jiang P, Jiang Y, Li C, Liu T, et al. (2017).MicroRNA-122a Regulates Zonulin by Targeting EGFR in Intestinal Epithelial Dysfunction. Cell Physiol Biochem.; 42: 848–58. doi: 10.1159/000478629 28641303

[24] Long YS, Deng GF, Sun XS, et al. (2011). Identification of the transcriptional promoters in the proximal regions of human microRNA genes. Mol Biol Rep. 38:4153–4157. doi: 10.1007/s11033-010-0535-y 21107707

[25] Qiu H, Liang D, Liu L, Xiang Q, Yi Z, Ji Y. (2020). A novel circulating miRNA-based signature for the diagnosis and prognosis prediction of early-stage cervical cancer. Technol Cancer Rese Treat. 19:1–7. doi: 10.1177/1533033820970667 33327867 PMC7750573

[26] Murata K, Furu M, Yoshitomi H, Ishikawa M, Shibuya H, Hashimoto M, et al. (2013). Comprehensive microRNA analysis identifies miR-24 and miR-125a-5p as plasma biomarkers for rheumatoid arthritis. PLoS One. 8:e69118. doi: 10.1371/journal.pone.0069118 23874885 PMC3715465

[27] Magro F, Langner C, Driessen A, Ensari A, Geboes K, Mantzaris GJ, et al. (2013). European consensus on the histopathology of inflammatory bowel disease. J. Crohns Colitis. 7:827–851. doi: 10.1016/j.crohns.2013.06.001 23870728

[28] Best W.R, Becktel J.M., Singleton J.W., et al. (1976). Development of a Crohn’s disease activity index. National Cooperative Crohn’s Disease Study Gastroenterology, 70 (3): 439–444. 1248701

[29] Schroeder KW, Tremaine WJ, Ilstrup DM. (1987). Coated oral 5-aminosalicylic acid therapy for mildly to moderately active ulcerative colitis. A randomized study. N Engl J Med. 317:1625–1629. doi: 10.1056/NEJM198712243172603 3317057

[30] Livak KJ, Schmittgen TD. (2001): Analysis of relative gene expression data using real-time quantitative PCR and the 2(-Delta Delta C (T)) method. Methods. 25:402–408. doi: 10.1006/meth.2001.1262 11846609

[31] Larabi A, Barnich N, Nguyen HTT. (2020). New insights into the interplay between autophagy, gut microbiota and inflammatory responses in IBD. Autophagy.16:38–51. doi: 10.1080/15548627.2019.1635384 31286804 PMC6984609

[32] Wawrzyniak M. & Scharl M. (2018). Genetics and epigenetics of inflammatory bowel disease. Swiss Med. Wkly 148, e00015. doi: 10.4414/smw.2018.14671 30378641

[33] Yarani R., Mirza A. H., Kaur S. & Pociot F. (2018). The emerging role of lncRNAs in inflammatory bowel disease. Exp. Mol. Med. 50, 1–14. doi: 10.1038/s12276-018-0188-9 30523244 PMC6283835

[34] Hur K, Kim SH, Kim JM. (2019). Potential Implications of Long Noncoding RNAs in Autoimmune Diseases. Immune Netw. Feb 25; 19(1):e4. doi: 10.4110/in.2019.19.e4 30838159 PMC6399094

[35] Li Y., Soendergaard C., Bergenheim F.H., Aronoff D.M., Milne G., Riis L.B. et al. (2018).COX-2-PGE2 Signaling Impairs Intestinal Epithelial Regeneration and Associates with TNF Inhibitor Responsiveness in Ulcerative Colitis. EBioMedicine. 36: 497–507. doi: 10.1016/j.ebiom.2018.08.040 30190207 PMC6197735

[36] Zhang C., Shu W., Zhou G., Lin J., Chu F., Wu H., et al. (2018). Anti-TNF- α Therapy Suppresses Proinflammatory Activities of Mucosal Neutrophils in Inflammatory Bowel Disease. Mediators Inflamm.; 2018:1–12. doi: 10.1155/2018/3021863 30595666 PMC6282128

[37] Olsen T., Rismo R., Gundersen M.D., Paulssen E.J., Johnsen K., et al (2016). Normalization of mucosal tumor necrosis factor-α: A new criterion for discontinuing infliximab therapy in ulcerative colitis. Cytokine.; 79:90–95. doi: 10.1016/j.cyto.2015.12.021 26775117

[38] Dovrolis N., Michalopoulos G., Theodoropoulos G.E., Arvanitidis K., Kolios G., Sechi L.A., et al. (2020). The Interplay between Mucosal Microbiota Composition and Host Gene-Expression is Linked with Infliximab Response in Inflammatory Bowel Diseases. Microorganisms.8:438. doi: 10.3390/microorganisms8030438 32244928 PMC7143962

[39] Xia L, Tan S, Zhou Y, Lin J, Wang H, Oyang L, et al. (2018). Role of the NFκB-signaling pathway in cancer. Onco Targets Ther. 11: 2063–2073. doi: 10.2147/OTT.S161109 29695914 PMC5905465

[40] Wang L, Walia B, Evans J, Gewirtz AT, Merlin D, Sitaraman SV. (2003).IL-6 induces NF-kappa B activation in the intestinal epithelia. J Immunol.;171:3194–201. doi: 10.4049/jimmunol.171.6.3194 12960348

[41] Woodford-Richens K, Bevan S, Churchman M, Dowling B, Jones D, Norbury CG, et al.(2000) Analysis of genetic and phenotypic heterogeneity in juvenile polyposis. Gut.46:656–60. doi: 10.1136/gut.46.5.656 10764709 PMC1727907

[42] Valmiki S., Ahuja V., Puri N., & Paul J. (2020). miR-125b and miR-223 Contribute to Inflammation by Targeting the Key Molecules of NFκB Pathway. Frontiers in medicine, 6, 313. doi: 10.3389/fmed.2019.00313 32039213 PMC6990118

[43] Liu Guangyao & Wang Yongkun & Zhang Mingran et al. (2019). Long non-coding RNA THRIL promotes LPS-induced inflammatory injury by down-regulating microRNA-125b in ATDC5 cells. International Immunopharmacology. 66. 354–361. doi: 10.1016/j.intimp.2018.11.038 30521964

[44] Song J, Liu D, Yin W (2022). lnc-THRIL and miR-125b relate to disease risk, severity, and imbalance of Th1 cells/Th2 cells in allergic rhinitis. Allergol Immunopathol (Madr). 1;50(3):15–23. 35527652 10.15586/aei.v50i3.528

[45] Wang JY, Xiao L, Wang JY. (2017). Posttranscriptional regulation of intestinal epithelial integrity by noncoding RNAs. *Wiley Interdiscip Rev RNA* 8: e1399. doi: 10.1002/wrna.1399 27704722 PMC5315612

[46] Hruskova V, Jandova R, Vernerova L, et al. (2016). MicroRNA-125b: association with disease activity and the treatment response of patients with early rheumatoid arthritis. Arthritis Res Ther. 2;18(1):124. doi: 10.1186/s13075-016-1023-0 27255643 PMC4890522

[47] Cheng NL, Chen X, Kim J, Shi AH, Nguyen C, Wersto R, et al.(2015). MicroRNA-125b modulates inflammatory chemokine CCL4 expression in immune cells and its reduction causes CCL4 increase with age. Aging Cell.;14:200–8. doi: 10.1111/acel.12294 25620312 PMC4364832

[48] Marina B, Marian H, Beiyan Z, Harvey F (2010). MicroRNA miR-125b causes leukemia. Proc Natl Acad Sci U S A. 14; 107(50): 21558–21563. doi: 10.1073/pnas.1016611107 21118985 PMC3003065

